# Short-Term Side Effects and SARS-CoV-2 Infection after COVID-19 Pfizer–BioNTech Vaccine in Children Aged 5–11 Years: An Italian Real-World Study

**DOI:** 10.3390/vaccines10071056

**Published:** 2022-06-30

**Authors:** Martina Capponi, Federica Pulvirenti, Bianca Laura Cinicola, Giulia Brindisi, Maria Giulia Conti, Giovanni Colaiocco, Giovanna de Castro, Cristiana Alessia Guido, Marzia Duse, Fabio Midulla, Anna Maria Zicari, Alberto Spalice

**Affiliations:** 1 Department of Maternal Infantile and Urological Sciences, Sapienza University of Rome, 00185 Rome, Italy; bianca.cinicola@uniroma1.it (B.L.C.); giulia.brindisi@uniroma1.it (G.B.); mariagiulia.conti@uniroma1.it (M.G.C.); giovanna.decastro@uniroma1.it (G.d.C.); cristiana.guido@uniroma1.it (C.A.G.); marzia.duse@fondazione.uniroma1.it (M.D.); fabio.midulla@uniroma1.it (F.M.); annamaria.zicari@uniroma1.it (A.M.Z.); alberto.spalice@uniroma1.it (A.S.); 2 Reference Centre for Primary Immune Deficiencies, Azienda Ospedaliera Universitaria Policlinico Umberto I, 00161 Rome, Italy; f.pulvirenti@policlinicoumberto1.it; 3 Department of Molecular Medicine, Sapienza University of Rome, 00185 Rome, Italy; 4 Department of Prevention and Public Health, Coordination of Vaccination Activities, Azienda Sanitaria Locale (ASL) Roma 2, 00157 Rome, Italy; giovanni.colaiocco@aslroma2.it

**Keywords:** COVID-19, SARS-CoV-2, children, vaccine, safety, side effects, immunization

## Abstract

Vaccination against COVID-19 is the most effective tool to protect both the individual and the community from this potentially life-threatening infectious disease. Data from phase-3 trials showed that two doses of the BNT162b2 vaccine were safe, immunogenic, and effective against COVID-19 in children aged 5–11 years. However, no surveys in real-life settings have been carried out in this age range. Here, we conducted a cross-sectional study to evaluate the short-term adverse reactions (ARs) and the rate of protection against infection of the BNT162b2 vaccine in children aged 5–11 years by the compilation of two surveillance questionnaires conceived using Google Forms. Five-hundred and ninety one children were included in the analysis. ARs were reported by 68.9% of the children, being mainly local. The incidence of systemic ARs, especially fever, was higher after the second dose. The incidence of infection after completing the immunization accounted for 13.6% of the children. COVID-19 symptoms reported were mild, with the exception of one case of pneumonia. Only 40% of infected participants needed to take medication to relieve symptoms, mostly paracetamol and NSAIDs, and none reported persistent symptoms. The Pfizer–BioNTech vaccine in children aged 5–11 years is safe and well tolerated. The mild clinical course of COVID-19 in immunized children confirmed the favorable risk–benefit ratio, encouraging parents to immunize their children.

## 1. Introduction

Severe acute respiratory syndrome coronavirus 2 (SARS-CoV-2), a new betacoronavirus identified as the causative agent of pneumonia cases of unknown etiology in late December 2019 in Wuhan [1], is responsible for coronavirus disease 2019 (COVID-19), declared a pandemic health emergency by the World Health Organization (WHO) in March 2020. Since then, around 500 million confirmed cases and almost 6.2 million deaths have been reported worldwide [2]. In Italy, 15.8 million cases have been diagnosed to date, of which 160,000 have died [3]. School-aged children account for a high percentage of COVID-19 cases [4]. The clinical presentation and severity of SARS-CoV-2 infections range from an asymptomatic course to severe pneumonia and multiorgan failure possibly leading to death [5]. In children, the clinical phenotype is mostly mild, with a high rate of asymptomatic or mildly symptomatic infections. Nevertheless, COVID-19 in children deserves special attention, due to their role in the transmission of the virus [6,7], including the highly contagious new variants [8,9]. Since the beginning of the epidemic in Italy, 3.5 million people aged < 19 years have been diagnosed with COVID-19, of which 17,300 required hospitalization, 388 were admitted in ICU, and 53 died [3]. Even if the disease is mostly milder in children than in adults, the emergence of occasional hospitalizations, the presence of vulnerable children in the population, and the rare but severe cases of multisystem inflammatory syndrome (MIS-C), as well as the possible long-term sequelae, have prompted research on the optimal treatments and prevention strategies [10,11]. Vaccination is the safest and most effective tool to protect both the individual and the community from serious or life-threatening infectious diseases. In this pandemic, an unprecedented effort has generated over 200 vaccine candidates in various stages of development, with over 50 candidate vaccines in human clinical trials and 18 in efficacy testing, with several vaccines reaching registration by health authorities. Seven vaccines have currently been approved by the WHO for use in children, and the United States was the first country to employ a messenger RNA (mRNA) technology-based vaccine in children aged >5 years (since November 2021) [12,13,14]. Pfizer–BioNTech (a lipid nanoparticle-formulated nucleoside-modified mRNA that encodes the receptor binding domain (RBD) of the SARS-CoV-2 spike protein [15]) received clearance for emergency use from the Food and Drug Administration (FDA) in December 2020 in people 16 years of age or older, with further extension to the age group 5–11 on 29 October 2021 [16]. Data from phase 3 trials showed that two 10-mcg doses of the BNT162b2 vaccine administered 21 days apart were safe, immunogenic, and 90.7% effective against COVID-19 in 5-to-11-year-old children. The most predominant side effects reported were injection-site pain (74%), fatigue (39%), and headache (28%) [17]. Although the efficacy and safety profiles of new vaccines have been extensively demonstrated in clinical trials, continuous surveillance in real-world conditions is needed. In this study, we enrolled children aged 5 to 11 years, immunized in two vaccination recruitment centers, with the aim to evaluate both the short-term side effects and the protection against infection of the COVID-19 Pfizer–BioNTech vaccine in children.

## 2. Materials and Methods

### 2.1. Study Design

To investigate the side effects in children in the age range 5–11 years following the administration of two doses of Pfizer–BioNTech (BNT162b2) mRNA vaccine, we conducted a cross-sectional study using a self-administered online survey. The study was carried out at the Vaccination Center of Policlinico Umberto I (Sapienza University of Rome). General and demographic data were collected, and vaccine-associated side effects following vaccination were evaluated. Inclusion criteria for the enrollment were being aged between 5–11 years and to have completed the immunization against COVID-19. Patients were recruited at AOU Policlinico Umberto I Pediatric Vaccination Center and at Santa Caterina Vaccination Center, ASL ROME 2, between 1 February and 28 February of 2022. All parents of children who agreed to participate signed the written informed consent form at enrollment. The surveillance surveys were developed through the compilation of two questionnaires conceived using Google Forms. The first questionnaire was designed to assess the prevalence of adverse reactions (ARs) following the first and the second dose of BNT162b2 vaccine, and the second one was designed to assess the incidence of SARS-CoV-2 infection in immunized participants and to characterize the clinical course of the disease. Both the questionnaires were developed after an extensive review of the literature with the aim to identify side effects post BNT162b2 vaccine administration and clinical manifestations and treatment of SARS-CoV-2 infection in children. The questionnaires were designed to have multiple sections and were accessible through a link sent by email. The first questionnaire was delivered two weeks after the administration of the second vaccine dose, while the second questionnaire was delivered 1 month after the second dose.

The first questionnaire included 38 questions divided into 7 sections. The first section (Q. 1–2) had an introductory part about the purpose of the study and it was designed to obtain the email address to promote the exchange of information between investigators and study participants. The second part (Q. 3–6) included sociodemographic information: child’s gender, child’s age, ethnic group, and region of residence. The third part (Q. 7–14) was formulated to investigate the presence of chronic diseases and allergies, highlighting the possible history of anaphylactic shock and/or severe reactions after previous vaccinations, and to verify a previous SARS-CoV-2 infection. Fourth and fifth sections (Q. 15–25), respectively, included information on local and systemic ARs after the first vaccine dose administration, whereas sixth and seventh sections (Q. 25–35) collected data on local and systemic ARs, respectively, following the second dose. Participants in the study were asked to qualify the type(s) of side effect(s) experienced, whether local and/or systemic(s) and to specify it by choosing, with multiple options, from a list of the main adverse effects reported in other studies. In addition we asked participants to report the timing of the ARs’ occurrence, and the need for medical assistance, hospitalization, and treatment after vaccination. We also investigated the impact of vaccination on limitations of daily activities. The second questionnaire was a 15-item tool designed to assess the possible rate and timing of COVID-19 infection in immunized children, the severity of COVID-19-related clinical manifestations, and the length of swab positivity. We also investigated if immunized children had symptoms after resolution of infection. The survey was sent four weeks after completing the immunization course.

### 2.2. Ethical Approval

The study protocol was approved by the Ethical Review Committee of Sapienza, University of Rome, Italy (Ref. 6689. Prot. 0254/2022, date March 2022). The study was performed in accordance with the Good Clinical Practice guidelines, the International Conference on Harmonization guidelines, and the most recent version of the Declaration of Helsinki.

### 2.3. Statistical Analysis

Based on the statistics available for the number of children in this age group among the whole country population (around 60 million), the sample size was calculated based on a 5% margin of error and a 95% confidence interval. Consequently, a sample size of 500 responses was determined to be sufficient for this study (www.raosoft.com, accessed on 1 May 2022). Demographics and clinical characteristics of the participants database were summarized with descriptive statistics. Sociodemographic and clinical variables were compared between the patients who developed ARs and AR-free patients and between those who had ARs after receiving a single vaccine dose vs. those who had ARs after receiving two doses. Statistical analysis was performed using frequency distributions. The χ^2^ test was used for categorical variables and the Mann U test was used for continuous variables. Statistical Package for Social Sciences version 15 (SPSS Inc., Chicago, IL, USA) was used for the analysis, with a significance level of *p* = 0.05.

## 3. Results

### 3.1. Study Participants

During the study period, 3125 doses of BNT162b2 were administered at the AOU Policlinico Umberto I Pediatric Vaccination Center and at Santa Caterina Vaccination Center, ASL ROME 2, of which 1406 (45%) were first doses. Overall, 591 vaccinated individuals accepted to voluntarily participate in the study. After removing 11 respondents (1.9%) without matching diaries for the first and the second dose, 579 children were included in the analysis.

Table 1 shows the demographic and vaccination characteristics of the enrolled subjects. Most of them were males (*n* = 306, 53%) with a mean age of 8.2 +/− 3 years. Caucasian children in the sample numbered 475 (82%). Most participants were referred to as being in good clinical condition, as scored by caregivers (*n* = 576, 99.5%). Seventy-one participants were reported as having an allergy (12.3%) and 28 (5%) as having chronic non-allergic diseases. The number of children who were infected by COVID-19 before study enrollment was 61 (10.5%). All patients received two doses of the BNT162b2 vaccine. Overall, almost 68.9% (*n* = 398) of the study participants experienced ARs, with no reports of vaccination-related serious adverse events, including myocarditis and anaphylaxis. These data are summarized in Table 1.

We then compared the children who experienced side effects with the children who did not according to age, sex, clinical status, presence of chronic diseases, previous infections with SARS-CoV-2, and past medical history of being positive for allergies (Table 2). We found that females were more likely to report ARs (51.5% vs. 37.6%, *p* = 0.002). Notably, we did not observe a higher incidence of ARs among individuals with a previous diagnosis of SARS-CoV-2 or those diagnosed with allergies (Table 2).

### 3.2. Reported Side Effects following the First or Second Doses

**Figure 1:**
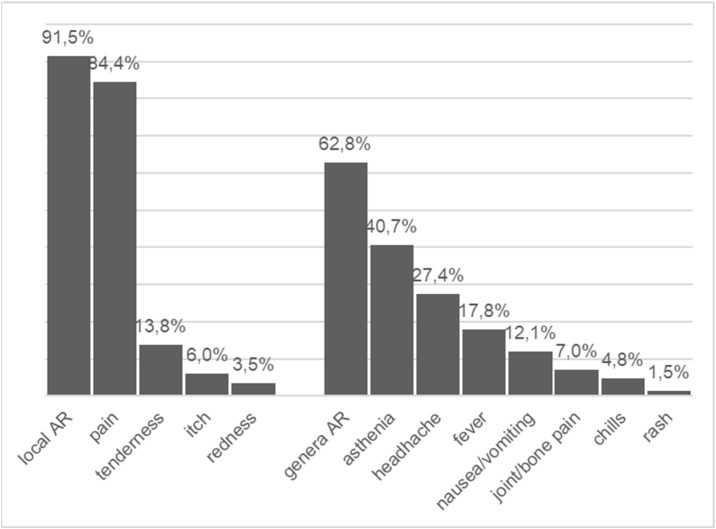
Proportion of local and systemic reactions in children with ARs following mRNA vaccine.

Among the 398 (69%) participants who reported at least one AR following administration of the first or the second vaccine dose, 91.5% (*n* = 364) reported local ARs and 62.8% (*n* = 250) systemic ARs. The most frequently reported local side effects were pain at the site of the injection (reported by 84.4%, *n* = 336) and tenderness (13.8%, *n* = 55). Asthenia (40.7%, *n* = 162) and headache (27.4%, *n* = 109) were the most commonly reported systemic ARs. Fever was reported by 17.8% (*n* = 71). Nausea or abdominal pain (12.1%, *n* = 48) and joint or bone pain (7.0%, *n* = 28) were rarely reported (Figure 1). Almost three out of four of the participants with ARs reported that the side effects started on the first day following vaccination, while one out of four noticed side effects starting at day 2 or later post vaccination (first or second dose) (Table 3).

We further categorized the children who reported ARs following vaccination into two groups: participants reporting ARs after receiving the first dose and those who reported ARs after the second dose. As seen in Table 4, ARs following the vaccine administration were reported by 57.3% of children (*n* = 332) after the 1st dose and by 59.6% (*n* = 345) of children after the second one. Among those with ARs, the incidence of local reactions was more frequent after the first dose (94.6% vs. 89.6%, *p* = 0.022), whereas systemic ARs’ incidence was significantly higher at the second dose (50.2% vs. 57.4%, *p* = 0.045). Pain at the site of the injection was the most frequently reported local AR (first dose: 89.5%, *n* = 290; second dose: 83.6%, *n* = 285, *p* = 0.039). Asthenia was the prevalent symptom reported (first dose: 34.1%, *n* = 113 and second dose: 36.9%, *n* = 127, *p* = 0.310). Headache and fever were more frequently reported after the second dose (18.7% *n* = 62 vs. 25.8% *n* = 89, *p* 0.044 and 7.9% *n* = 26 vs. 16.6%, *n* = 57, *p* < 0.0001). Other systemic ARs such as joint and muscular pain, a rash, and shivers were rarely reported. Notably, those with ARs after the second vaccine dose were limited in daily activities more frequently (first dose 12.6% vs. second dose 35.0%, *p* < 0.0001). The previous history of COVID-19 infection and being affected by allergy were not related with having ARs after the first or second dose of vaccine (Table 5).

### 3.3. SARS-CoV-2 Infection after Immunization

From the 579 participants who completed the first questionnaire, 293 (50.6%) responded to the second questionnaire (age: mean 8.1 +/− 2,00, females 46%). Forty (13.6%) participants were reported to have been infected by SARS-CoV-2 (age: mean 8.2 +/− females 48.7%), and 31 (77.5%) reported to be symptomatic. Symptoms of COVID-19 were mild in all but one child, with only one case of pneumonia reported. Most reported symptoms were nasal stuffiness/nasal discharge (*n* = 24, 77%), fever (*n* = 17, 55%), asthenia (*n* = 14, 45%), a dry cough (*n* = 13, 42%), headache (*n* = 12, 39%), and joint/muscular pain (*n* = 8, 26%, Table 5. The duration of the infections was reported to have been within one week in 50% of patients (*n* = 20), and 16 patients needed medication to relieve COVID-19 symptoms, mostly paracetamol and NSAIDs. Only the patient who developed pneumonia needed to be hospitalized. Three patients reported symptoms even after infection resolution: one reported asthenia and nasal stuffiness for less than two weeks, one reported a cough and asthenia lasting less than one week, and one dyspnea and diarrhea for one week.

## 4. Discussion

Vaccination extended across age groups appears to be the most effective tool for controlling the COVID-19 pandemic in an effort to gain herd immunity as soon as possible.

In Italy, after approval from the European Medicines Agency (EMA), the Italian Drug Agency (AIFA) started vaccination in the 5–11 age group on 16 December 2021; however, by the end of March 2022, vaccination coverage was still unsatisfactory: only 3.8% of children had received one dose and 33.1% had received two doses (ISS) [3]. The main social and clinical problems that still hinder vaccination programs include parental hesitation due to concern about potential side effects, the diffidence generated by the high rate at which vaccines have been developed, and the lack of data on the long-term effects of immunization in children. Moreover, high anxiety has been generated by very rare serious events following immunization, including reports of myocarditis [18] and anaphylaxis with the mRNA vaccines, and atypical forms of thrombotic events with thrombocytopenia and Guillain–Barré syndrome with adenovirus vector vaccines [19,20]. Therefore, it is essential to try to overcome the vaccine hesitancy that mainly results from a lack of knowledge about the relative benefit–risk ratio of vaccination, through continuous monitoring of and updates about the safety and effectiveness of COVID-19 vaccines, with the aim of improving adherence to vaccination. Moreover, strengthening the reassurance and acceptance of COVID-19 vaccines among the public is important in an effort to control the spread of viral infection, limiting the emergence of new variants [21].

To contribute to this need, here we conducted the first study on the incidence of adverse events following the administration of Pfizer/BioNTech (BNT162b2) anti-COVID-19 vaccines in children between the age of 5–11 years old. We recorded that ARs were reported in nearly seven out of ten study participants. However, the reported ARs were mild, with no severe adverse reactions being reported. The observed incidence of ARs was similar to those observed in a previous study on children aged 12–18 years [22], and higher than reported in adults, confirming the observation that younger people experience post-immunization adverse effects more frequently than older ones [23]. We confirm the predisposition of females to develop adverse reactions to the vaccine more frequently than males [22,24,25,26]. Unlike other reports [22,25,26], in our study the history of a previous diagnosis of SARS-CoV-2 was not a predictor for adverse effects after vaccination. This may have been influenced by the fact that by including only children who completed the vaccine course with two doses of the BNT162b2 vaccine, the SARS-CoV-2 infection should have occurred at least 4 months prior to enrollment. Moreover, the prevalence of COVID-19 infection recorded in this study was lower than reported in Europe in the same period [27] since pediatric acute COVID-19 has likely been underestimated given the milder presentation of the disease and the testing paradigms. The underrating of infections may explain the missing correlation between exposure to COVID 19 before the administration of the vaccine and adverse events.

As expected from the results of clinical trials and real-world surveys [17,22,23,24,25,28], local reactions were more frequent, while systemic ARs occurred with a lower incidence. Among local reactions, injection site pain was the most frequently reported AR, while among systemic events, asthenia and headache were the most commonly reported, confirming the results of pivotal clinical trials in both children aged 5 to 11 years [17] and in adults/adolescents [23,28,29]. The published literature shows that most side effects associated with the COVID-19 vaccine are mild to moderate and limited to the first two days after vaccination [29,30]. Here we confirm the data, observing that ARs started on the 1st day following vaccination in almost three out of four of the participants. In our study, systemic ARs’ incidence, especially fever, was significantly higher at the second dose, supporting clinical trials data and observation in real-world studies on different age ranges [30,31,32]. This increased reactogenicity in those who received two vaccine doses could be explained by an increased vaccine-induced immunogenicity possibly leading to a more intense inflammatory response after the second dose in comparison to the first [26].

The risk of myocarditis after vaccination in children aged 5 to 11 years is a main topic of concern. Although a precise estimate of the risk of such conditions is currently unknown, it is expected to be lower in this age range in comparison to adolescents and young people [33]. Furthermore, the available data on a few cases of post-immunization myocarditis with a mild course show that the benefits of mRNA vaccination largely exceed these very rare risks [33,34]. According to this observation, with the limitations of a short-term study, no cases of myocarditis were recorded in our study as in the clinical trial [17]. Although extremely rare, few cases of MIS-c are described in adults after vaccination [34]; in our study, as in others carried out in children, including clinical trials in this age group [17], no cases of MIS-c were reported after vaccination.

The assessment of the effectiveness of the BNT162b2 vaccine, defined as the ability to prevent SARS-CoV-2 infection, disease, or transmission [35], is an issue of great relevance, and so is the effect of immunization on infection course mitigation. Even if not designed for this purpose due to the lack of a control group of non-immunized children, here we were able to measure the incidence and characterize the clinical course of COVID-19 infection in immunized children 4 weeks after completing the vaccination.

Differently from infection in adults, the clinical presentation and severity of SARS-CoV-2 in children is mostly mild, with a high rate of asymptomatic or mildly symptomatic infections. However, severe cases of multisystem inflammatory syndrome (MIS-C), as well as the possible long-term sequelae also in children otherwise healthy, have been described [10,11]. Recently, in a meta-analysis including >9300 children/adolescents (0–19 years) with documented SARS-CoV-2 infection, the mean proportion of asymptomatic children was 13%, with the majority of symptoms being a fever (63%), cough (34%), nausea/vomiting (20%), diarrhea (20%), dyspnea (18%), nasal symptoms (17%), rashes (16%), fatigue (16%), abdominal pain (15%), conjunctivitis (11%), and pharyngeal erythema (9%). Severe COVID-19-related manifestations were also reported, including Kawasaki-like signs (13%), neurological symptoms (12%), and MIS-C [35].

The role of vaccination in reducing SARS-CoV-2 symptoms is still to be clarified. Previous randomized trials have demonstrated a risk reduction for symptomatic and severe COVID-19 within the first months after BNT162b2 vaccination in both children and adults. In a large placebo-controlled trial, the efficacy in preventing symptomatic COVID-19 of the two-dose vaccine course after one month accounted for 95 percent for individuals >16 years of age [23], 100 percent for individuals aged 12 to 15 years [28], and 91 percent for individuals aged 5 to 11 years [36]; this latter rate is close to that observed in our study. It is unclear if this higher frequency of infection in immunized children in the 5–11 age range is related to a lower effectiveness in comparison to their older counterparts or to a general reduced vaccine effectiveness against the more contagious Omicron variant, which dominated soon after the introduction of the vaccine for the younger children. However, vaccination still appears to be protective against COVID-19-associated hospitalizations in this age group, even in the context of Omicron prevalence, although the estimate of effectiveness is uncertain because hospitalizations are uncommon [37,38,39,40]. More recently, a large US case–control study conducted during the Omicron variant’s predominance, estimated the Odds Ratio for symptomatic infection 2 to 4 weeks after the second vaccine dose as being 0.40 both in children and in adolescents [41].

The length of infections was reported to be within one week in almost all the participants, and only 40% needed medication to relieve COVID-19 symptoms, mostly paracetamol and NSAIDs. The incidence of persistent symptoms after infection resolution was low, confirming again the efficacy of the immunization in the prevention of a severe COVID-19 course. The main limitation of this study was the short-term evaluation of adverse effects. Indeed, long-term surveillance in the general population will be required to investigate possible future effects.

## 5. Conclusions

In conclusion, to the best of our knowledge, this is the first surveillance study conducted in children between 5 and 11 years old based on real-life data.

Our data confirm the results of the clinical trials. Predominantly, local adverse effects were observed, and the general effects were mild and of short duration. No serious adverse events were recorded. The clearly favorable risk–benefit ratio must therefore encourage the most reluctant parents to start vaccination in order to extend the low coverage in children and to reduce the risk of a serious course of COVID-19. Longitudinal studies are needed to confirm this acceptable long-term safety profile. Finally, the control of COVID-19 infections contributes to the reduction in the spread of the virus in the general population, preventing the emergence of new variants.

## Figures and Tables

**Table 1:** Demographic and clinical characteristics of enrolled children.

Characteristics of Participants		Study Participants (*n* = 579)	
		*n*	%
Age	5–7 years	243	42.0%
	8–11 years	335	58.0%
Sex	Male	306	52.8%
	Female	273	47.2%
Ethnic group	Caucasian	475	82.0%
	Non-Caucasian	104	18%
Good health condition	No	2	0.40%
	Yes	576	99.5%
Allergy	No	508	87.7%
	Yes	71	12.3%
Chronic diseases	No	550	95.0%
	Yes	29	5.0%
Diagnosed previously with COVID-19	No	518	89.5%
	Yes	61	10.5%
Report of at least one AR after vaccination	No	181	31.1%
	Yes	398	68.9%

**Table 2:** Univariate analysis of the participants who presented with ARs compared to those without ARs following BNT162b2 vaccination.

Participants’ Characteristics	Children with ARs (*n* = 398)		Children without ARs (*n* = 181)		*p*-Value
Age < 8 years, *n* (%)	150	(37.7)	93	(51.4)	0.003
Sex (female), *n* (%)	205	(51.5)	68	(37.6)	0.002
Ethnicity (Caucasian), *n* (%)	326	(81.9)	149	(82.3)	0.905
Good clinical status, *n* (%)	396	(99.5)	180	(99.4)	0.938
Chronic Disease, *n* (%)	20	(5.0)	8	(4.4)	0.661
Allergy, *n* (%)	50	(12.6)	20	(11.0)	0.605
History of COVID-19, *n* (%)	42	(10.6)	19	(10.5)	0.984

**Table 3:** Timing, needs for medication or medical assistance, and disability due to post-immunization ARs.

	Children with ARs (*n* = 398)	
	*n*	%
Timing of ARs		
First day	210	70.0%
Second day	84	28.0%
Third day	6	2.0%
Needed medication to mitigate ARs	119	29.9%
Visiting a physician due to ARs	4	1.0%
Hospitalization due to ARs	1	0.3%
Unable to perform normal daily activities	140	35.2%

**Table 4:** Absolute numbers and relative frequencies of adverse reactions after the first and the second dose of BNT162b2. Characteristics of patients are also included.

Adverse Reactions	ARs after Single Dose, *n* = 332 (57.3%)		ARs after Two Doses, *n* = 345 (59.6%)		*p*-Value
Local ARs, *n* (%)	314	(94.6)	309	(89.6)	0.022
Pain	290	(89.5)	285	(83.6)	0.039
Tenderness	40	(12.3)	38	(11.1)	0.79
Itch	15	(4.6)	20	(5.9)	0.402
Redness	10	(3.1)	6	(1.8)	0.307
Systemic ARs, *n* (%)	166	(50.2)	198	(57.4)	0.045
Asthenia	113	(34.1)	127	(36.9)	0.31
Headache	62	(18.7)	89	(25.8)	0.044
Fever (>37.5 °C)	26	(7.9)	57	(16.6)	<0.0001
Joint pain	22	(6.7)	19	(5.5)	0.629
Abdominal pain	15	(4.5)	37	(10.8)	0.002
Chills	11	(3.3)	10	(2.9)	0.826
Rash	1	(0.3)	5	(1.5)	0.102
Needed medication, *n* (%)	70	(21.3)	92	(26.8)	0.089
Visiting a physician due to ARS, *n* (%)	0	(0)	1	(0.3)	0.969
Hospitalization due to ARs, *n* (%)	0	(0)	1	(0.3)	0.969
Unable to perform daily activities, *n* (%)	42	(12.6)	121	(35)	<0.0001
Allergy, *n* (%)	42	(11.6)	32	(12.8)	0.4723
History of COVID19, *n* (%)	35	(11)	38	(10)	0.9016

**Table 5:** SARS-CoV-2 infection in children after two doses of immunization.

SARS-CoV-2 Infections Parameters Considered	Children with SARS-CoV-2 Infection *n* = 40	
Days from first dose, *n* (%)		
14–30 days	8	(21)
31–40 days	10	(26.3)
41–50 days	11	(28.9)
>50 days	9	(23.7)
**COVID-19-related symptoms, *n* (%)**	31	(77.5)
Nasal stuffiness/nasal discharge	24	(77)
Fever	17	(55)
Asthenia	14	(45)
Cough	13	(42)
Headache	12	(39)
Joint/muscular pain	8	(26)
Restlessness/insomnia	4	(13)
Vomiting/diarrhea	2	(6)
Pneumonia	1	(3)
Anosmia	1	(3)
**Duration of symptoms, *n* (%)**		
<5 days	19	(61.3)
5–7 days	10	(32.2)
unknown	2	(6.4)
Duration of COVID-19, *n* (%)		
<5 days	3	(7.5)
5–7 days	17	(42.5)
8–15 days	15	(37.5)
<15 days	1	(2.5)
Unknown	4	(10)

## Data Availability

All data generated or analysed during this study are available upon request from the corresponding author if required.
